# Retromer Dependent Recycling of the Wnt Secretion Factor Wls Is Dispensable for Stem Cell Maintenance in the Mammalian Intestinal Epithelium

**DOI:** 10.1371/journal.pone.0076971

**Published:** 2013-10-09

**Authors:** Reinoud E. A. de Groot, Henner F. Farin, Marie Macůrková, Johan H. van Es, Hans C. Clevers, Hendrik C. Korswagen

**Affiliations:** 1 Hubrecht Institute, Royal Netherlands Academy of Arts and Sciences and University Medical Centre Utrecht, Utrecht, The Netherlands; 2 Department of Cell Biology, Faculty of Science, Charles University, Prague, Czech Republic; Institut Curie, France

## Abstract

In *C. elegans* and *Drosophila*, retromer mediated retrograde transport of Wntless (Wls) from endosomes to the trans-Golgi network (TGN) is required for Wnt secretion. When this retrograde transport pathway is blocked, Wls is missorted to lysosomes and degraded, resulting in reduced Wnt secretion and various Wnt related phenotypes. In the mammalian intestine, Wnt signaling is essential to maintain stem cells. This prompted us to ask if retromer mediated Wls recycling is also important for Wnt signaling and stem cell maintenance in this system. To answer this question, we generated a conditional *Vps35* ^*fl*^ allele. As Vps35 is an essential subunit of the retromer complex, this genetic tool allowed us to inducibly interfere with retromer function in the intestinal epithelium. Using a pan-intestinal epithelial Cre line (*Villin-CreERT2*), we did not observe defects in crypt or villus morphology after deletion of *Vps35* from the intestinal epithelium. Wnt secreted from the mesenchyme of the intestine may compensate for a reduction in epithelial Wnt secretion. To exclude the effect of the mesenchyme, we generated intestinal organoid cultures. Loss of *Vps35* in intestinal organoids did not affect the overall morphology of the organoids. We were able to culture *Vps35* ^*∆/∆*^ organoids for many passages without Wnt supplementation in the growth medium. However, Wls protein levels were reduced and we observed a subtle growth defect in the *Vps35* ^*∆/∆*^ organoids. These results confirm the role of retromer in the retrograde trafficking of Wls in the intestine, but show that retromer mediated Wls recycling is not essential to maintain Wnt signaling or stem cell proliferation in the intestinal epithelium.

## Introduction

The mammalian intestinal epithelium is a rapidly self-renewing tissue. Stem cells endow the intestine with its proliferative capacity. Intestinal stem cells reside at the bottom of invaginations of the intestinal epithelium; the crypts of Lieberkühn. The intestinal stem cells are characterized by expression of Lgr5 [1], they are actively cycling and give rise to cells that proliferate in the transiently amplifying (TA) compartment of the crypt [2]. Cells move up from the TA compartment and differentiate in the villus domain. The villus epithelium consists of enterocytes, goblet cells and enteroendocrine cells. Paneth cells are differentiated cells that reside at the bottom of the crypt. The Paneth cells are part of the stem cell niche that supports the intestinal stem cells [3]. Various signaling pathways - such as the Wnt, Notch and EGF signaling cascades - are required to maintain intestinal homeostasis [2], but Wnt signaling is of particular importance because it drives proliferation and is essential for stem cell maintenance. 

Wnt signaling in intestinal stem cells is activated by Wnt ligands that are expressed in the Paneth cells and cells in the intestinal mesenchyme [4]. Wnt signaling is enhanced by R-spondin, which is the ligand of the stem cell marker Lgr5 [5]. It is essential that a fine balance of Wnt pathway activity is maintained in the intestine, as overactivation of Wnt signaling results in adenoma formation and ultimately leads to cancer [6]. 

Detailed knowledge has accumulated about the mechanism of Wnt signal transduction in Wnt receiving cells, but the mechanism of Wnt secretion has only recently been uncovered (reviewed in 7,8). Wnt protein is produced in the ER and lipid modified by the O-acyltransferase Porcupine [9,10]. Wnt follows the secretory pathway to the Golgi apparatus where it associates with Wntless (Wls), a transmembrane protein that is essential for Wnt secretion [11–13]. Wls escorts Wnt from the Golgi to the plasma membrane where Wnt is released. Importantly, studies in *C. elegans*, *Drosophila* and mammalian tissue-culture cells have shown that Wls needs to be retrieved back to the trans-Golgi network (TGN) to maintain Wnt secretion. This retrieval route involves AP-2 and clathrin mediated endocytosis of Wls from the plasma membrane [14–16] and transport from endosomes to the TGN, a retrograde trafficking step that is mediated by the retromer complex [14,15,17–20]. In the absence of a functional retromer complex, Wls is retained in the endosomal system and degraded in lysosomes. As a result, less Wls is available in the Golgi to mediate Wnt secretion, leading to various Wnt signaling related phenotypes [14,15,18–22].

The retromer complex is a multi-protein complex that mediates transport of membrane proteins from endosomes to the TGN. Retromer cargo proteins include the cation-independent mannose-6-phosphate receptor (CI-MPR), Sortilin, the polarity protein Crumbs and Wls (reviewed in 23). Vps35 is the central cargo-binding subunit of the retromer complex and loss of Vps35 strongly reduces Wnt secretion in *C. elegans, Drosophila* and mammalian tissue culture cells [14,18–21]. 

Retromer mediated recycling of Wls is required for Wnt signaling in invertebrate model systems, but the *in vivo* role of this retrieval pathway has not been tested in mammalian Wnt signaling. We generated a floxed allele of *Vps35* to conditionally interfere with retromer function in the murine intestinal epithelium. We investigated the effect of *Vps35* deletion *in vivo*, and in a recently established intestinal organoid culture system. We show that Vps35 is required to maintain Wls protein levels in intestinal cells, but growth of intestinal organoids was only mildly affected. This suggests that retromer mediated recycling of Wls is dispensable in the mammalian intestinal epithelium in steady state conditions.

## Materials and Methods

### ES cell targeting and generation of mouse strains

**Figure 1:**
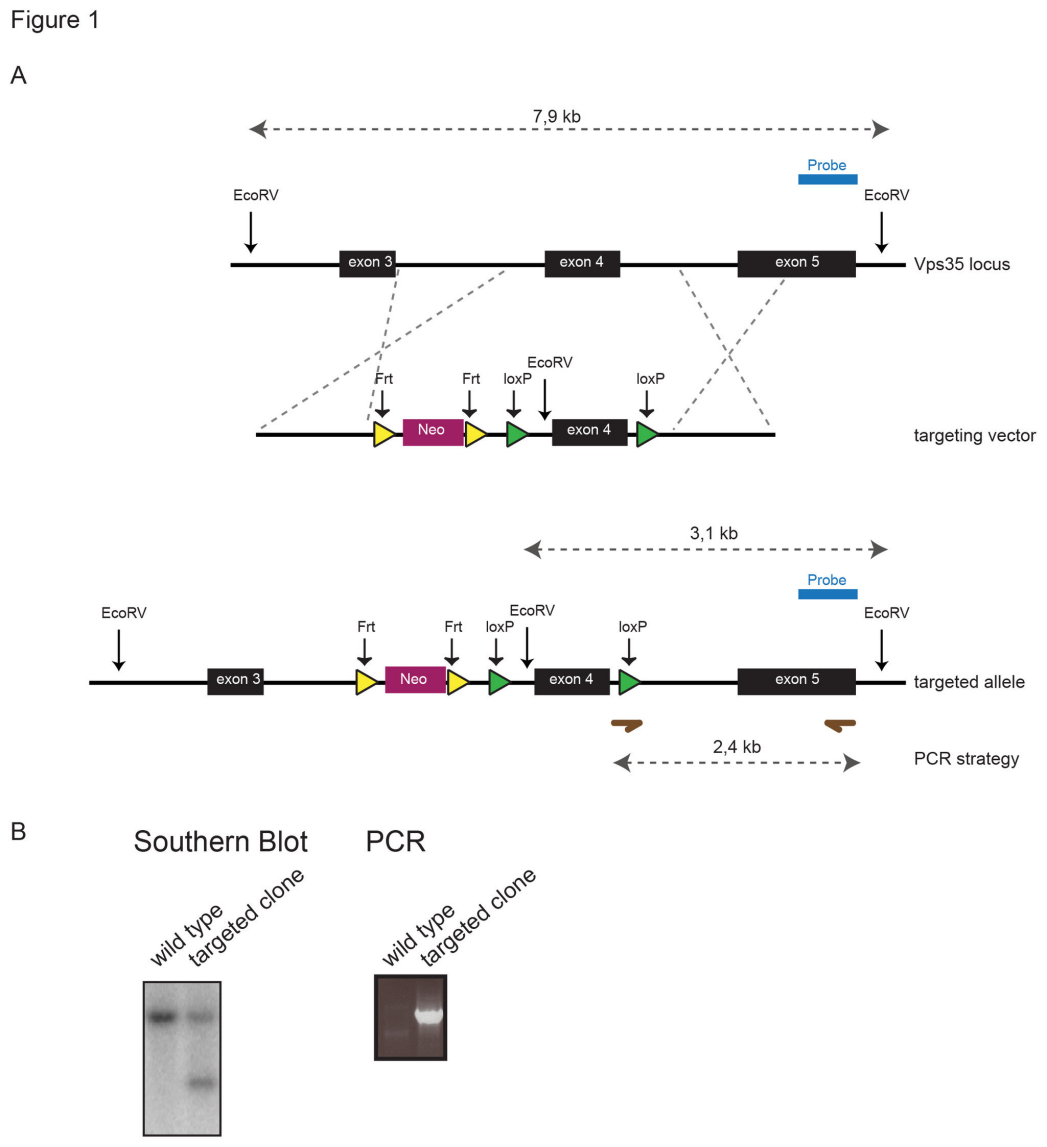
Targeting strategy to generate a conditional *Vps35* allele. (A) LoxP sites were introduced in the third and fourth introns of the *Vps35* gene by homologous recombination. (B) Southern blot and PCR analysis showing correct targeting of selected ES cell clone.

Conditional Vps35 mice were generated by homologous recombination in embryonic stem cells using a targeting construct that is schematically depicted in Figure 1A. Exon 4 and flanking homology arms were PCR amplified from 129/Ola derived DNA to generate a targeting construct. The linearized construct was transfected into male 129/Ola derived IB10 embryonic stem cells by electroporation (800V, 3F). Recombinant targeted ES cell clones were selected in medium supplemented with G418, TK was used as counter selection. Clones were screened by Southern blotting and confirmed by PCR analysis. Positive clones were injected into C57BL/6 blastocysts. The neomycin selection cassette was excised *in vivo* by crossing the mice to the FLPeR deleter strain. *Vps35* ^*fl*^ mice were crossed to the *Villin-CreERT2* strain [24]. Recombination *in vivo* was induced in 4 week old mice by intraperitoneal 4-OHT injection (5 mg 4-OHT, dissolved in 200 l sunflower oil). Mice were sacrificed 3 days, 1 week, 4 weeks and 8 weeks after Cre induction. Histology and immunohistochemistry was performed as described in [25]. The animal experiments were approved by the Animal Experimentation Committee of the Royal Academy of Arts and Sciences (protocol number HL06.1010). 

### Intestinal organoid culture

Mouse organoids were derived from isolated crypts of the proximal small intestine of a *Vps35* ^*fl/fl*^;*Villin-CreERT2* mouse as described in [26]. The organoids were maintained in ENR culture medium in a drop of Matrigel (BDBiosciences) as described [26]. The ENR culture medium consists of advanced Dulbecco’s modified Eagle medium/F12 supplemented with penicillin/streptomycin, 10 mmol/L HEPES, 1xGlutamax, 1xB27 (Life Technologies), 1 mmol/L N-acetylcysteine (Sigma), 50 ng/mL murine recombinant EGF (Peprotech), R-spondin1 (conditioned medium, 10% final volume) and Noggin (conditioned medium, 10% final volume). The conditioned media were produced using HEK293T cells stably transfected with HA-mouse Rspo1-Fc (gift from Calvin Kuo, Stanford University) or after transient transfection with mouse Noggin-Fc expression vector. Advanced Dulbecco’s modified Eagle medium/F12 supplemented with penicillin/streptomycin, 10 mmol/L HEPES, and 1xGlutamax was conditioned for 1 week. Wnt3a conditioned medium was produced using stably transfected L cells after 1 week of conditioning in medium containing 10% fetal bovine serum. To induce *Vps35* deletion, 4-OHT (Sigma, 0.5 M/L) was added to the culture medium for 12 hours. Organoid growth was quantified by scoring the number of buds that had developed 5 days after passaging. 

### RT-PCR and qPCR

Organoids were dissolved in TRIzol (Life technologies), RNA was isolated from organoids using an RNAeasy kit (Qiagen) and cDNA was generated using the RT-II kit (Invitrogen) using oligo dT primers. Primers sequences used for RT-PCR:

Vps35: CTGTTGGCTCTCCTTCATCAG; AACTGCACTACTTGGAGGTC,
Cdx2: GTACACAGACCATCAGCGGC; CCACCCCATCCAGTCTCACT,
Lgr5: TGCCATCTGCTTACCAGTGTTGT; ATTCCGTCTTCCCACCACGC,
Lysozyme: GAGACCGAAGCACCGACTATG; CGGTTTTGACATTGTGTTCGC,
Mucin2: GAACGGGGCCATGGTCAGCA; CATAATTGGTCTGCATGCC.

qPCR was performed using the iQ SYBR green reagent in a MiIQ real-time PCR system (Biorad). Relative expression was calculated using the ΔΔCt method relative to Ywhaz expression. Primer sequences used for qPCR: 

Axin2: TGACTCTCCTTCCAGATCCCA; TGCCCACACTAGGCTGACA, Ywhaz: TGCAACGATCTACTGTCTCTTTTG; CGGTAGTAGTCACCCTTCATTTTCA.

### Western blot analysis of organoid proteins

Organoids were washed twice in ice cold PBS and taken up in Laemli sample buffer. Samples were boiled for 5 minutes prior to SDS-PAGE and Western blotting following standard procedures. The following antibodies were used for detection: anti-Wls (ab72385-500, Abcam), anti-Vps35 (ab10099-100, Abcam), anti-alpha-Tubulin (DM1A, Sigma), anti-mouse-HRP (GE Healthcare), anti-chicken-HRP (Abcam), anti-rabbit-HRP (GE Healthcare).

## Results

**Figure 2:**
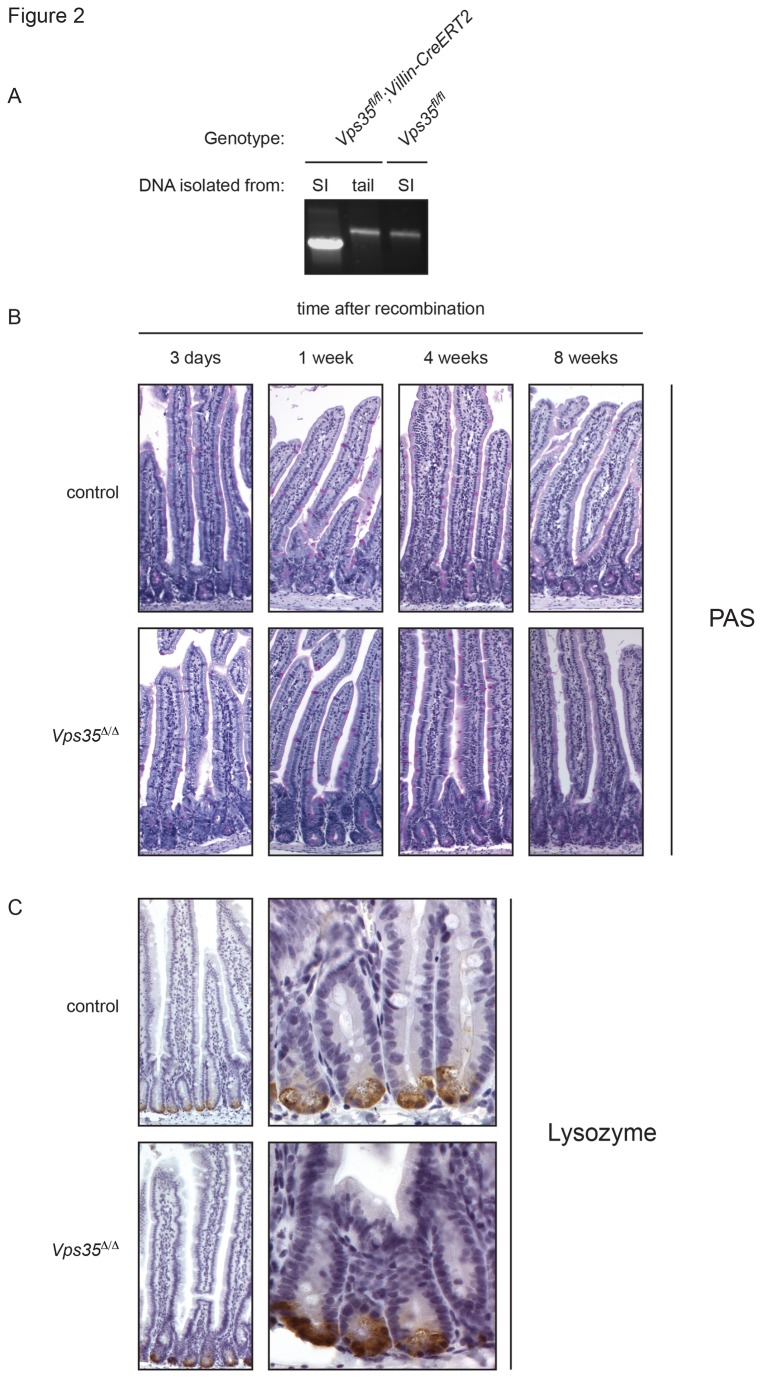
Knockout of *Vps35 in vivo*. (A) PCR analysis using primers that anneal outside *Vps35* exon 4 shows deletion of exon 4 from genomic DNA isolated from small intestinal epithelium (SI) of 4-OHT induced *Vps35* ^*fl/fl*^ (control) or *Vps35* ^*fl/fl*^ *; Villin-CreERT2* mice (*Vps35^∆/∆^*). Histological analysis of *Vps35* knockout intestine showed no defects in crypt-villus morphology. Intestine sections were Periodic Acid Schiff (PAS) stained (B) and immunohistochemistry was performed to stain Lysozyme (C).

Since loss of retromer function is embryonic lethal [27] and we aimed to specifically investigate the role of Vps35 in the intestinal epithelium, we generated a conditional *Vps35* ^fl^ allele to inducibly delete *Vps35* in the intestinal epithelium of 4 week old mice. We introduced loxP sites in the third and fourth intron of *Vps35* by homologous recombination in mouse embryonic stem cells. We crossed the *Vps35* ^*fl*^ mice to animals carrying a CreERT2 transgene driven by the *Villin* promotor (Figure 1A, B). This allowed us to inducibly delete *Vps35* in the intestinal epithelium using intraperitoneal 4-Hydroxytamoxifen (4-OHT) injections. We confirmed that recombination occurred in the intestinal epithelium by PCR on genomic DNA obtained from intestinal epithelial cells (Figure 2A). We examined the intestines of the mice 3 days, 1 week, 4 weeks and 8 weeks after 4-OHT injection. We performed periodic acid Schiff (PAS) staining and immunohistochemistry to detect the Paneth cell marker Lysozyme, but we found no qualitative differences in crypt or villus morphology of *Vps35* ^*∆/∆*^ mice compared to control littermates (Figure 2B, C). This result indicates that Vps35 is dispensable for intestinal homeostasis in adult mice. 

**Figure 3:**
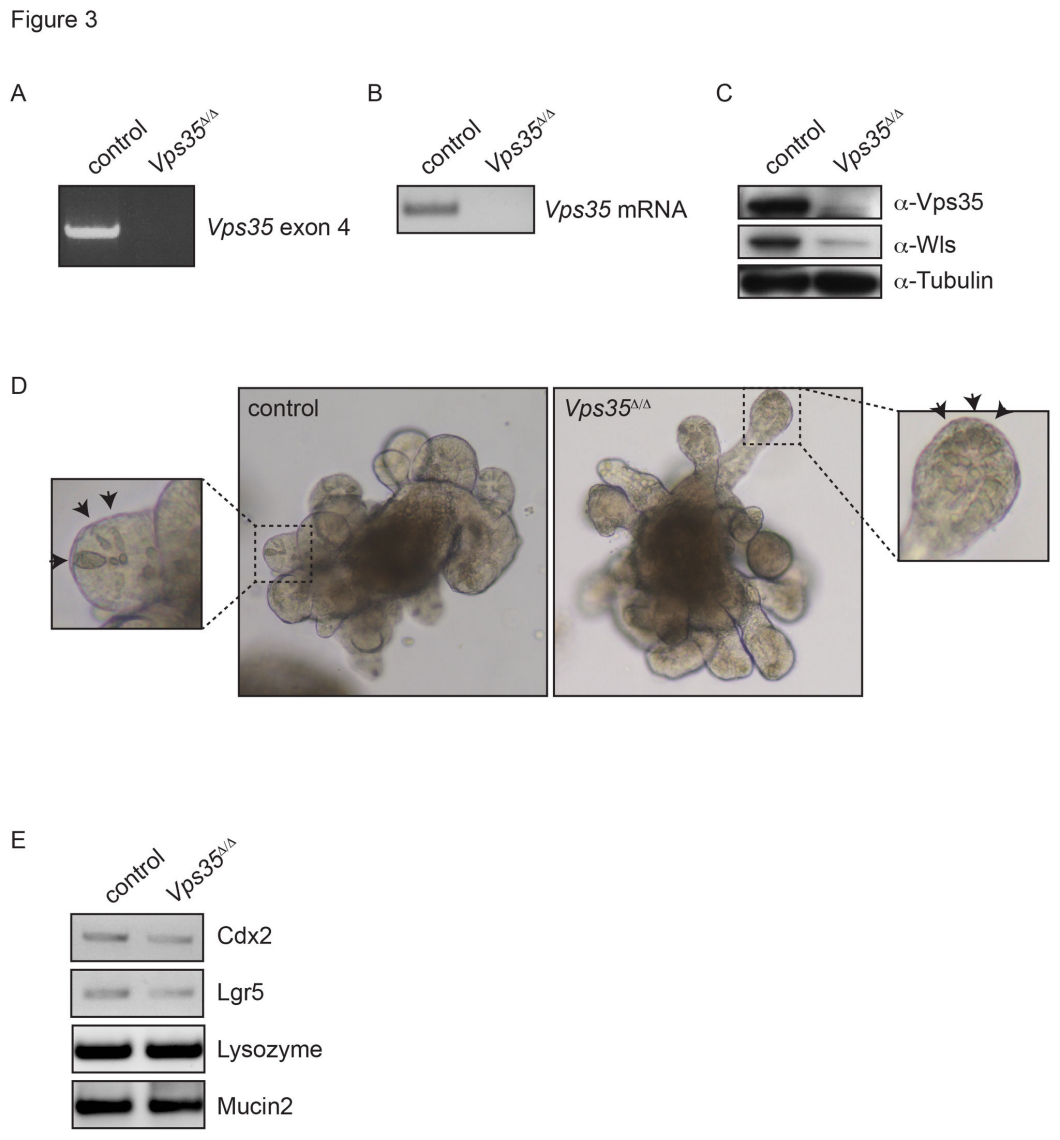
Knockout of *Vps35* in intestinal organoids. Intestinal organoids were obtained from a *Vps35* ^*fl/fl*^ *; Villin-CreERT2* mouse and treated with 0.5 μM 4-OHT for 12 hours (*Vps35^∆/∆^*), or control treated (control). (A) PCR analysis of genomic DNA from *Vps35* knockout organoids shows complete deletion of exon 4 of *Vps35* *in* *vitro*. (B) RT-PCR shows absence of *Vps35* exon 4 from mRNA of *Vps35* ^*∆/∆*^ organoids. (C) Western blot analysis shows absence of Vps35 protein and reduced Wls protein levels in *Vps35* ^*∆/∆*^ organoids. (D) *Vps35* ^*∆/∆*^ organoids show normal morphology, Paneth cells are indicated by arrowheads. (E) RT-PCR analysis of molecular markers of differentiated intestinal cells and intestinal stem cells.

It was recently shown that Wnt ligands secreted from the mesenchyme of the intestine can compensate for loss of *Wnt3* from the intestinal epithelium [4]. We reasoned that these mesenchyme derived Wnt ligands may also compensate for a reduction in Wnt secretion induced by loss of *Vps35* from epithelial cells. In order to investigate the effect of loss of *Vps35* specifically in the intestinal epithelium, without the influence of the surrounding mesenchyme, we derived intestinal organoids from the *Vps35* ^*fl/fl*^ *; Villin-CreERT2* mice. Intestinal organoids can develop form single Lgr5(+) stem cell and consist solely of epithelial cells. Organoids form crypt-like buds that contain stem cells and Paneth cells as well as villus-like domains that contain differentiated cells [26]. We induced recombination *in vitro* by addition of 4-OHT to the culture medium. Using PCR analysis, we found that the recombination of the *Vps35* ^*fl/fl*^ allele was complete (Figure 3A, B). Western blot analysis showed that Vps35 protein was absent form the *Vps35* ^*∆/∆*^ organoids. Importantly, we found that Wls protein levels were strongly reduced (Figure 3C), demonstrating that Vps35 is required to maintain Wls levels in the murine intestinal epithelium. These results are consistent with the current model of Wls trafficking and for the first time show that retromer dependent trafficking is required for Wls stability in the mouse. 

We did not observe any major morphological changes in the phenotype of the *Vps35* ^*∆/∆*^ organoids. Paneth cells were visible at the tips of the buds (Figure 3D) and reverse transcriptase PCR (RT-PCR) analysis showed that the *Vps35* ^*∆/∆*^ organoids express the intestine marker *Cdx2*, the stem cell marker *Lgr5*, the Paneth cell marker *Lysozyme* and the goblet cell marker *Mucin2* (Figure 3E). We were able to culture the *Vps35* ^*∆/∆*^ organoids for many passages (>30) in standard intestinal organoid culture medium that contains EGF, Noggin and R-spondin (ENR medium). In contrast, deletion of *Wnt3* from intestinal organoids causes a characteristic ‘pointy crypt’ phenotype and a loss of Paneth cells in the buds [4]. Furthermore, *Wnt3* ^*∆/∆*^ organoids cannot be maintained in regular ENR medium, but need Wnt supplemented in the medium for continuous culturing [4]. We conclude that deletion of Vps35 does not affect the gross morphology or block differentiation or proliferation of intestinal organoids.

**Figure 4:**
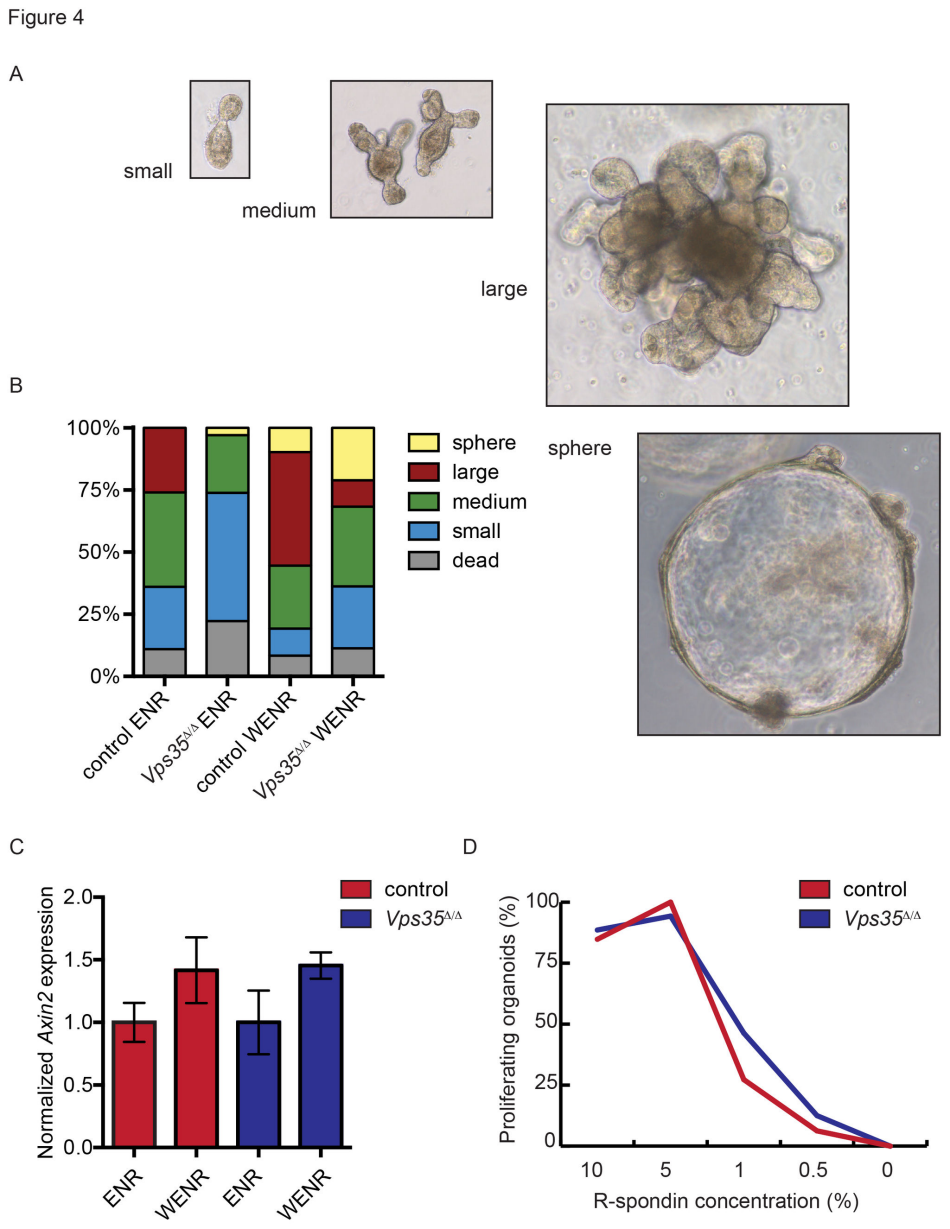
*Vps35* ^*∆/∆*^ organoids show a growth defect but are competent to respond to Wnt signaling. (A) Growth was quantified by scoring the organoids in categories based on the number of buds the organoids had produced 5 days after passaging. (B) *Vps35* ^*∆/∆*^ organoids show reduced proliferation compared to control organoids. This could not be completely rescued by Wnt3a supplemented in the medium (ERN: small intestine organoid medium, containing EGF, R-Spondin, Noggin, WENR: ERN medium supplemented with 30% Wnt3a conditioned medium). (C) *Vps35* ^*∆/∆*^ organoids can respond to Wnt signaling as assayed by Axin2 qPCR (data are represented as mean ± SD, n=3). (D) Percentage of growing organoids cultured in ENR medium with varying R-spondin concentrations.

 Next, we quantified the growth of *Vps35* ^*∆/∆*^ organoids and compared it to control organoids. We categorized the organoids based on the number of crypt-like buds that had developed five days after passaging and used this as a proxy for the growth rate (Figure 4A). We found that *Vps35* ^*∆/∆*^ organoids consistently had fewer buds compared to control organoids. However, we found that this reduction in growth rate can only be partly attributed to a reduction in Wnt3 secretion because supplementation of exogenous Wnt3a in the medium did not fully rescue this growth defect (Figure 4B). These results show that expansion of *Vps35* ^*∆/∆*^ organoids is reduced through both Wnt dependent and Wnt independent effects.

We found that the *Vps35* ^*∆/∆*^ organoids are competent to respond to Wnt3a, since both control and *Vps35* ^*∆/∆*^ organoids formed spheroid structures upon Wnt3a stimulation, a phenotype that is characteristic for Wnt pathway activation in intestinal organoids [3]. Furthermore, quantitative PCR (qPCR) analysis showed that Wnt3a stimulation induced a similar upregulation of the Wnt target gene Axin2 in control and *Vps35* ^*∆/∆*^ organoids (Figure 4C). 

 Finally, we investigated the dependence of *Vps35* ^*∆/∆*^ organoids on the Wnt signaling agonist R-spondin. R-spondin is the ligand for Lgr5 and Lgr4 and forms an essential component of the culture medium of intestinal organoids [5]. We titrated the R-spondin concentration in the medium, but we found no difference in survival rates between control and *Vps35* ^*∆/∆*^ organoids cultured in different R-spondin concentrations (Figure 4D).

## Discussion

Retromer dependent recycling of Wls is essential for efficient Wnt secretion in *C. elegans, Drosophila* and mammalian tissue culture cells [14,17–20], but the *in vivo* role of Wls recycling in mammalian Wnt signaling has not been tested. In the murine intestine, Wnt signaling is required for proliferation and stem cell maintenance. We therefore investigated whether the retromer complex is required for stem cell maintenance and tissue homeostasis in the intestine.

We generated a floxed allele to inducibly delete *Vps35* from the intestinal epithelium of adult mice. We did not observe defects in the morphology of the intestine of these mice. To circumvent potential effects of Wnt secretion from the surrounding mesenchyme, we derived *Vps35* ^*fl/fl*^ intestinal organoids. Deletion of *Vps35* in these organoids showed that Vps35 is required to maintain Wls protein levels in the intestinal epithelium. This confirms the role of retromer in retrieving Wls from the endosomal-lysosomal degradative pathway in intestinal cells. Surprisingly, the *Vps35* ^*∆/∆*^ organoids could be cultured for many passages and showed no morphological defects. Loss of *Vps35* did not affect the ability of the organoids to respond to Wnt signals or the dependence on R-spondin. However, the *Vps35* ^*∆/∆*^ organoids showed a reduced growth rate compared to control organoids. This proliferation defect cannot be completely rescued by supplementation of Wnt3a in the medium, indicating that *Vps35* controls organoid growth through both Wnt dependent and Wnt independent effects.

Why does loss of Vps35 and the resulting reduction in Wls protein levels cause only a subtle proliferation defect in the mouse intestine? In *C. elegans*, loss of the retromer complex mainly affects Wnt signaling processes that act over a relatively long distance, such as neuroblast migration and the establishment of neuronal polarity [21,22]. These Wnt signaling processes require the formation of long-range Wnt concentration gradients and are therefore dependent on efficient Wnt secretion. In contrast, loss of retromer function does not significantly affect Wnt signaling processes that take place between neighboring cells [21]. Also in the *Drosophila* wing imaginal disc, loss of retromer mainly affects the expression of high threshold Wnt target genes, while low threshold target genes such as *Distalless* are normally expressed [18–20]. Taken together, these studies show that loss of retromer reduces, but not eliminates Wnt secretion in worms and flies. Stem cells in intestinal organoids require stimulation by Wnt proteins that are secreted from neighboring Paneth cells [3]. In analogy with *C. elegans*, this short range Wnt signaling may be less sensitive to loss of *Vps35*. In addition, Wnt signaling in intestinal stem cells is amplified by R-spondin, a ligand that acts through the Lgr5 receptor [5]. This amplification mechanism may also explain why the intestinal stem cells are relatively insensitive to a reduction in Wnt secretion. Finally, it has been shown that Wls is a Wnt target gene in the mouse [28]. By stimulating the expression of Wls, secretion of mammalian Wnt proteins may be less dependent on retromer mediated recycling of Wls. 

The subtle growth defect that we observed could not be fully rescued by supplementation of Wnt3a in the culture medium. Missorting of other retromer cargos such as the CI-MPR, Sortilin or the polarity protein Crumbs, may negatively influence organoid growth [23]. It was recently shown that Lgr5 undergoes retrograde traffic from endosomes to the TGN in Human Embryonic Kidney (HEK) cells. Therefore, Lgr5 itself may be a retromer cargo in these cells [29] and potentially in the intestinal stem cells. However, the fact that the *Vps35* ^*∆/∆*^ organoids were equally dependent on R-Spondin argues against a critical role of retromer in Lgr5 regulation. We were unable to detect endogenous Lgr5 protein in organoid lysates by Western blot (data not shown), so we could not confirm if Lgr5 is a retromer cargo in intestinal cells.

In our experimental approach, we investigated the role of Vps35 in Wnt signaling and stem cell maintenance in intestinal homeostasis of juvenile and adult mice. In these conditions, retromer mediated recycling of Wls is dispensable. There may be situations, for example during embryonic development or during regeneration after injury, which require enhanced levels of Wnt secretion, and may therefore be more dependent on retromer mediated retrograde transport of Wls. For example, Wnt5a signaling is essential for intestinal tube elongation during development and regeneration of the colon after tissue injury [30,31]. It will be interesting to determine if *Vps35* ^*∆/∆*^ mice have defects in recovery from injury, or if deletion of *Vps35* during development of the intestine causes defects.

In summary, we show that the retromer complex is required to maintain high Wls protein levels in intestinal epithelial cells, which is in agreement with the current model of Wls trafficking. Proliferation and the maintenance of stem cells in organoids are however minimally affected by loss of *Vps35*. The mouse strain that carries the floxed *Vps35* allele will be a valuable tool to study retromer function during development and regeneration of the intestine as well as in other tissues and in different biological contexts.
